# Automatic localisation of landmark points in CMR images using a cyclic motion mask

**DOI:** 10.1186/1532-429X-14-S1-T9

**Published:** 2012-02-01

**Authors:** Jorge Novo Buján, Robert Merrifield, Lichao Wang, Guang-Zhong Yang

**Affiliations:** 1 Computing, University of A Coruña, A Coruña, Spain; 2 Hamlyn Centre for Robotic Surgery, Imperial College London, London, UK

## Background

Many cardiac segmentation algorithms require the input of an initial estimate of the position and orientation of the heart. This work presents a method for automatically localising the LV/RV centres and junction points in short axis mid ventricular CMR images. The technique uses a novel cyclic motion mask that removes the influence of static tissue.

## Methods

For each pixel in a short axis TrueFISP image, the negative changes to the signal intensities over the cardiac cycle are summed to produce a motion map that highlights the contraction of the ventricles. A second motion map that emphasises the expansion of the ventricles is created by summing the positive pixel changes. A Gaussian filter is applied to both of the maps which are intersected to produce a cyclic motion mask that emphasises image regions that undergo both contraction and expansion. The motion mask is modulated with the original image to highlight the LV and RV. A Hough transform is applied to identify the centre of the LV. The filtered image is reformatted to polar coordinates about the centre of the LV. A profile corresponding to the sum of pixel intensities in each row is analysed to find the minimum corresponding to the horizontal line of the LV myocardium. All pixels below this line are excluded. A Gaussian filter is applied and the centre of the RV is identified as the maximum pixel value. The filtered image is then reformatted to polar coordinates about the centre of the RV. The LV/RV junction points are identified by analysing a profile of pixel intensities in each column.

## Results

**Figure 1:**
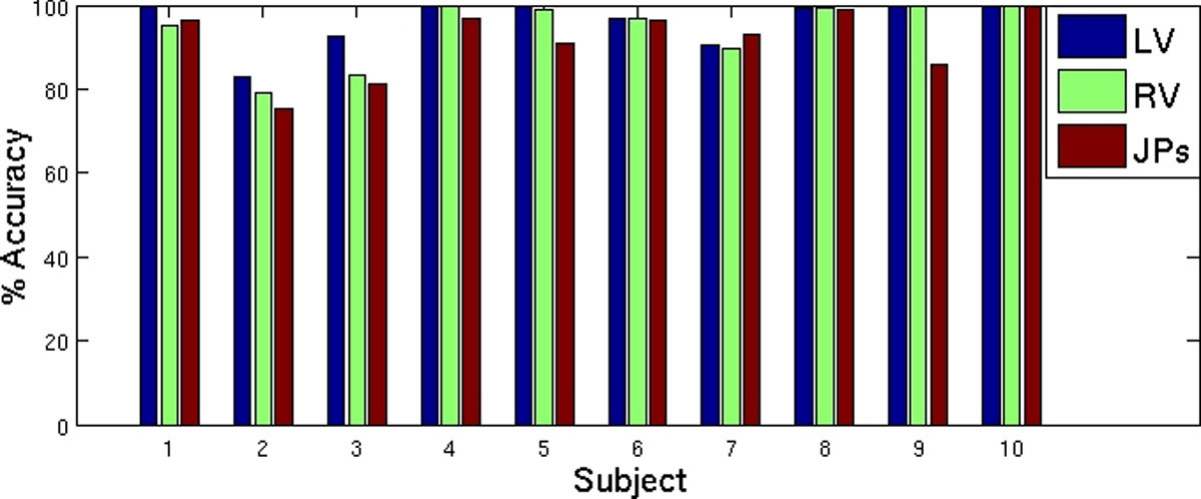
A histogram of the accuracy of the technique for each of the 10 subjects.

For testing the methodology, 10 asymptomatic subjects were imaged using a TrueFISP sequence on a 1.5T Siemens Sonata Scanner. 5 mid ventricular short slices were obtained with a slice thickness of 7.0 mm and an in-plane resolution between 1.1mm and 2.0mm. Between 20 and 25 cine frames were acquired for each slice resulting in the acquisition of 1125 images in total. The identified centres and junction points were considered correct if they lay within 10mm of manually identified points. The results demonstrate a good success rate in global terms (Table 1) and also for each patient (Figure 1).

**Table 1:** Accuracy of the automated detection of the LV and RV centres and junction points for all 10 subjects.

	Left Ventricle	Right Ventricle	Junction Points
Accuracy	96.24%	94.16%	91.53%

## Conclusions

In this work, we present a novel method for detecting key cardiac landmark points in short axis mid ventricular CMR images. This work has the potential to provide the initial estimate of the cardiac position and orientation to a wide range of cardiac segmentation algorithms.

## Funding

This paper was funded by the charity for prevention of heart disease and stroke CORDA and by the Consellería de Industria, Xunta de Galicia, Spain (Grant contract 10TIC009CT).

